# The diversity and evolution of electric organs in Neotropical knifefishes

**DOI:** 10.1186/s13227-022-00194-5

**Published:** 2022-04-01

**Authors:** Isabelle E. Bray, Ilham J. J. Alshami, Tetsuhiro Kudoh

**Affiliations:** 1 Biosciences, College of Life and Environmental Sciences, University of Exeter, Exeter, UK; 2 Department of Fisheries and Marine Resources, College of Agriculture, University of Basrah, Basrah, Iraq

**Keywords:** Gymnotiformes, Electric fish, Electric organ, Myogenic electric organ, Neurogenic electric organ, Electrocytes, Pear-shaped nerve cords, PSNCs

## Abstract

The Gymnotiformes, also known as the South American or Neotropical knifefishes, include the strongly electric *Electrophorus electricus* and many other weakly electric species. These fish possess specialised electric organs that are able to release electric discharges into the water, for electrolocation and communication, and sometimes for predation and defence. All Gymnotiform species possess a myogenic electric organ (mEO) derived from the muscle tissue, and members of the Apteronotidae family uniquely possess a neurogenic electric organ (nEOs) derived from the nervous tissue. A mEO may consist of ‘Type A’ electrocytes that develop within the tail muscle (for example, in *Apteronotus leptorhynchus*), or ‘Type B’ electrocytes that develop below the tail muscle (for example, in *Brachyhypopomus gauderio*). In this review, we discuss the diversity in the anatomy, electric discharge and development of electric organs found in different Gymnotiform species, as well as the ecological and environmental factors that have likely contributed to this diversity. We then describe various hypotheses regarding the evolution of electric organs, and discuss the potential evolutionary origin of the nEO: a pair of nerve cords that are located on either side of the aorta in *B. gauderio*, and which may have expanded and developed into a nEO in the Apteronotidae family during its evolution from a common ancestral species. Finally, we compare potential Gymnotiform phylogenies and their supporting evidence.

## Introduction

Electric fish are unique models for studying the evolution of both electric and sensory organs. Numerous different linages have evolved these organs independently; the taxonomic diversity of electric fish is so profound, in fact, that Darwin specifically named them as an important example of convergent evolution [12]. Amongst the most well-studied are the Gymnotiformes, an order of teleosts commonly known as the South American or Neotropical knifefishes [32, 49]. These species possess a characteristic anguilliform body, with a short head and trunk region and a long tail; they do not have dorsal, adipose, pelvic, nor caudal fins, but instead swim using a remarkably long ventral fin that extends throughout the length of the tail [9, 33, 38, 47]. The Gymnotiformes can be divided into three major groups: Gymnotidae (the Electrophorus and Gymnotus genera), Rhamphichthyoidea (the Rhamphichthyidae and Hypopomidae families) and Sinusoidea (the Sternopygidae and Apteronotidae families) [52].

Like other electric fish, the Gymnotiformes can be categorised as either strongly electric (the electric eel, *Electrophorus electricus*—a member of the Gymnotidae group) or weakly electric (the vast majority of species) [38]. Weakly electric species produce discharges for electrolocation, during which they sense objects in their environment by detecting distortions in the electric fields they produce, and electrocommunication, during which they communicate with other individuals of the same, or sometimes different, species (for example, during mate recognition and selection) [7, 8, 44]. Conversely, strongly electric species produce discharges to incapacitate prey, and defend against being predated on themselves [38].

As with all electric fish, the Gymnotiformes are able to generate electric discharges through the use of a specialised electric organ [44]. Most species possess a myogenic electric organ (mEO) derived from the muscle tissue, but members of the Apteronotidae family (commonly known as the ghost knifefishes) possess a neurogenic electric organ (nEO) derived from the nervous tissue [31]. The current-producing cells of the electric organ are called the electrocytes [27]. These function largely through the production of action potentials [54], although this process has been much better characterised for the mEO than it has for the nEO.

In this review, we set out what is known about both the mEO and the nEO in the Gymnotiformes, in terms of anatomy, electric discharge and development. We then consider the ecological factors that have shaped their evolution, examine a wide range of theories regarding how they have evolved, and finally, discuss potential Gymnotiform phylogenies and their supporting evidence.

It is worth noting some additional concepts that come into play (although they are not focuses of this review). In addition to producing electric discharges, electric fish must be able to sense them, which they do through electroreceptors that are distributed over their skin [5, 37]. Each individual uses these for the detection of perturbations of their own electric discharges, as well as the detection of discharges of other individuals [44]. Additionally, the shape of an individual discharge may vary between species; for example, the discharge of one species may consist of a single phase (a monophasic discharge) [38], while that of other species may consist of two or three phases (a diphasic or triphasic discharge) [18, 31]. The pattern with which these individual discharges are released may also vary; for example, wave-type species release discharges as continuous, oscillating signals, whereas pulse-type species release discrete discharges that are separated by pauses [17, 44]. Two non-Gymnotiform families are referred to during the discussion of electric organ evolution: the Mormyridae (the elephantfishes) and the Malapteruridae (the electric catfishes).

### The myogenic electric organ (mEO)

The majority of electric fish possess a mEO derived from the muscle tissue [15, 31]. Since a multitude of studies have been performed for this organ, it will be discussed both as a whole and in the context of specific Gymnotiform species.

### The anatomical diversity of the mEO in Gymnotiformes

In the Gymnotiformes, the mEO is a long structure running throughout the tail, spanning the majority of the length of the body [31]. It is composed of horizontal layers of electrocytes, and each layer consists of two rows running along the length of the organ—one on the left side of the body, and one on the right [1, 19, 45]. Electrocytes in different layers are also arranged in vertical columns. We have characterised this structure for the bluntnose knifefish, *Brachyhypopomus gauderio* [1], and other studies have done so for *E. electricus* [45]; these findings show that the number of layers present varies between species. *E. electricus* is unique in that adults possess three electric organs instead of one: a main organ, a Sach’s organ, and a Hunter’s organ [24, 57]. These organs dominate the posterior 80% of the animal, while the viscera are crowded into the anterior 20% [24]. The main organ extends from just behind the viscera along most of the tail, where it gives rise to the Sach’s organ which extends along the remaining portion of tail and the smaller Hunter’s organ is located below the other two [45].

Two types of myogenic electrocyte have been identified in the Gymnotiformes. ‘Type A’ electrocytes originate from within the tail muscle, between muscle fibres [31]. These have been observed in *Eigenmannia virescens*, *Sternopygus macrurus* and the Tbrown ghost knifefish, *Apteronotus leptorhynchus*. ‘Type B’ electrocytes originate from a distinct germinative zone located below the tail muscle [31]. These have been observed in *E. electricus*, *B. gauderio*, *Brachyhypopomus pinnicaudatus,* and various *Gymnotus* and *Rhamphichthys* species [1, 31, 45]. Though Type A electrocytes have been reported to possess more similarities to muscle fibres than Type B ones [31], the two types do not appear to be drastically different. We have extensively studied the mature electrocytes of *B. gauderio*, which are extraordinarily large compared with other cells such as myocytes [1]. We found that although their centres are devoid of cellular structures, there is a peripheral accumulation of organelles and cytoskeleton near the plasma membrane—notably numerous nuclei as syncytium, microtubules, mitochondria and actin filaments [1]. Similar features have been observed in the myogenic electrocytes of other Gymnotiformes [27, 45], although their shape differ; for example, they are cigar-shaped in *S. macrurus and E. virescens*, cuboidal in *Bracyhypopomus* and *Gymnotus* species, and flattened in *E. electricus* [61].

Since myogenic electrocytes are specialised for membrane excitability rather than contraction (which is explained further below), they have increased levels of proteins involved in the former and are deficient in proteins involved in the latter, when compared to the muscle fibres from which they are derived [45]. However, in the Gymnotiformes they have still been found to contain muscle-specific proteins, such as dystrophin, desmin and two forms of actin [1, 27, 45].

### The electric discharge of the mEO in Gymnotiformes

The Gymnotiformes are generally weakly electric, although *E. electricus* is able to produce both a strong discharge thanks to the main organ and a weak discharge thanks to the Hunter’s and Sach’s organs [7, 38]. The shape of the electric discharge produced by the mEO tends to change during development. In the vast majority of species, the larval discharge is monophasic, and some (including *E. electricus*, *Gymnotus cylindricus*, various *Eigenmannia* species and a small *Brachyhypopomus* species) still possess monophasic discharges as adults [31, 38, 46]. However, most have more complex adult discharge types; for example, some *Brachyhypopomus* species, *Gymnotus* species and members of the Rhamphichthyidae family have a biphasic adult discharge [7, 30, 38, 46].

The electric discharge from the mEO represents the summated activity of many electrocytes, synchronously transforming action potentials into external signals [54]. This process has been particularly well-characterised for the monophasic discharge produced by *E. electricus* [58]. The electrocytes are polarised, with their transmembrane proteins unevenly distributed between an ‘innervated’ membrane on the posterior side and a ‘non-innervated’ membrane on the anterior side. Specialised spinal motor neurons activate the electrocytes by releasing the neurotransmitter acetylcholine [27]. This binds to acetylcholine receptors in the innervated membrane, which become permeable to sodium and potassium ions [58]. The movement of these ions into the cell depolarises the innervated membrane, causing its normally negative potential to become positive [19, 36, 58]. This triggers voltage-gated sodium channels in the innervated membrane to open, and the resulting influx of sodium ions into the cell in turn amplifies depolarisation, creating a positive feedback loop. This results in the formation of an action potential on the innervated membrane. Following this, the innervated membrane becomes repolarised and regains its negative resting potential as the voltage-gated sodium channels close, and ions exit the cell through leak channels [58]. While an action potential is formed on the innervated membrane, the non-innervated membrane retains its negative resting potential thanks to the activity of sodium potassium ATPase pumps and various ion channels within it [2, 19, 26]. This results in a potential difference across the cell, and a net electric current from the innervated membrane to the non-innervated membrane [58]. Much like batteries, the electrocytes are arranged in series and in parallel, which allows their currents to be summated [6, 14]. This current is channelled along the body towards the head by the insulating connective tissue surrounding the electrocytes, and discharged into the environment as a single ‘head positive’ phase [14].

Though the process described above is largely conserved among electric fish species possessing a mEO, it can vary slightly to produce different discharge shapes [14, 44]. For example, for a biphasic discharge, current may first travel towards the head to form a head-positive phase (as described above), then towards the tail to form a ‘head negative’ phase. For this to occur, both the posterior side and the anterior side of each electrocyte must possess the same transmembrane proteins, so that the posterior side can act as the innervated side during the head-positive phase, after which the anterior side can act as the innervated membrane during the head-negative phase [14, 44].

### The development of the mEO in Gymnotiformes

In Gymnotiformes possessing Type A electrocytes, the mEO develops within the tail muscle [31]: *A. leptorhynchus* and *E. virescens* are unique in that they initially develop a larval organ, which produces an electric discharge for a short period before degenerating and being replaced by a second electric organ in a separate location at the adult stage. In *E. virescens*, this adult electric organ is another mEO, but in *A. leptorhynchus* it is a nEO. On the contrary, *S. macrurus* produces only a single mEO during development. In *E. virescens* and *S. macrurus*, the adult mEO continues to grow longitudinally along the tail until it reaches its full extension [31].

**Fig. 1:**
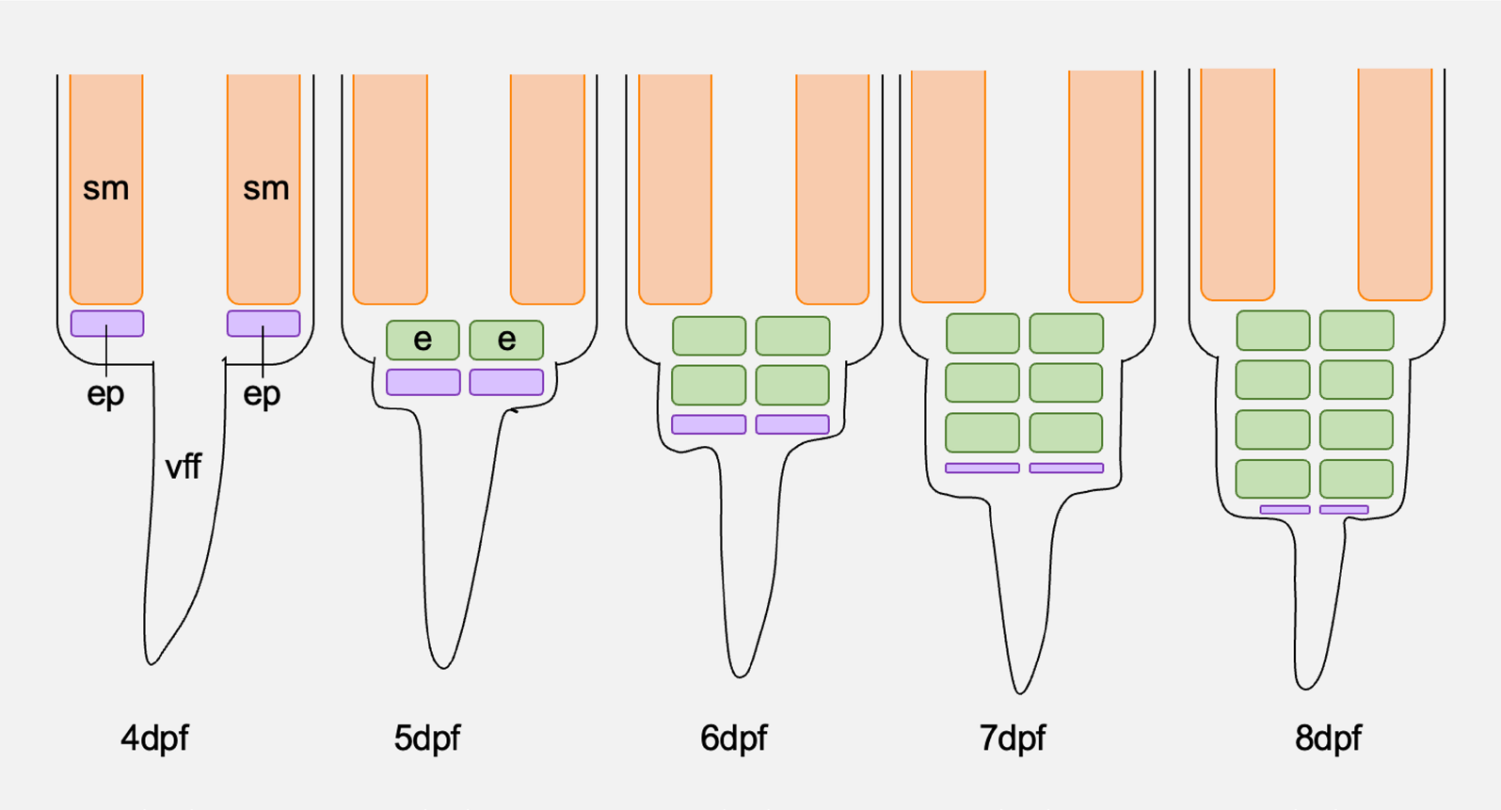
A transverse diagrammatical representation of the development of the myogenic electric organ (mEO) in *Brachyhypopomus gauderio* [1]. A pair of dense cell masses constituting an electric organ primordium (ep) develop on the ventral side of the somite muscle (sm) at around 3.5 to 4 days post-fertilisation. During the following days, these cell masses move downwards into the ventral fin fold (vff), and approximately each day a pair of electrocytes (e) are formed behind them, in the space between them and the somite muscle. By 8 days post-fertilisation, approximately four rows of electrocytes have formed within the ventral fin fold, and the cell masses have significantly decreased in size [1]

In Gymnotiformes possessing Type B electrocytes, the mEO develops beneath the tail muscle [31]. Development is similar in all of these species, in that several rows (or layers) of mature electrocytes are sequentially formed parallel to the germinative region from which they originate [1, 31]. However, *E. electricus* is unique in that this germinative region continues to give rise to new rows of electrocytes for a relatively long time, presumably in order to produce all three of the electric organs [31]. The early stages of the main organ can be detected in a 10-mm-long hatchling and those of the Hunter’s organ in a 140-mm-long juvenile, but the Sach’s organ appears only very late during development [31]. We recently performed an extensive study of the germinative region in *B. gauderio* [1]. We found that the mEO begins to develop below the tail muscle at 4.5 days post-fertilisation, then grows downwards into the ventral fin. We also observed the formation of a pair of dense cell masses at the bottom of the tail muscle at 3.5 days post-fertilisation. Because these are located at the leading edge of the developing mEO and contain cells with features typical of undifferentiated embryonic cells, we suggested that they represent an electric organ primordium, and deposit precursor cells that differentiate into electrocytes. After it appears, this primordium gradually moves downwards through the ventral fin, and each day between 6.5 and 9.5 days post-fertilisation, a pair of electrocytes are formed behind it as it does so. Once the whole mEO has been formed, the primordium gradually decreases in size [1]. This developmental process is illustrated by Fig. 1. A mEO primordium has also been identified in larval stages of *E. electricus*, in which it was termed the ‘electromatrix’ [45]. The fact that this appears morphologically similar to the *B. gauderio* primordium, and results in a similar layered pattern in the newly formed mEO, suggests that a mEO primordium is conserved among *B. gauderio*, *E. electricus* and other Gymnotiformes that possess Type B electrocytes [1].

An area of mEO development that is vital to consider is the origin of the electrocytes, and there are multiple contrasting hypotheses concerning how they arise. This has been studied in the Gymnotiformes through the process of regeneration—observations following adult tail amputation have described the regeneration of numerous tissue types, including the mEO [28, 29, 53, 55]. It has been shown that during regeneration in *S. macrurus*, mature muscle fibres fuse with one another before transdifferentiating into electrocytes (in part by disassembling their myofibrils) [41, 61]. Further studies suggesting the presence of undifferentiated myoblasts (the precursor cells that give rise to muscle fibres) around the regenerating mEO [55] indicate that the process of transdifferentiation involves the dedifferentiation of muscle fibres to myoblasts, followed by the redifferentiation of these myoblasts to electrocytes. However, more recent studies point towards an alternative hypothesis: that electrocytes in Gymnotiformes develop from distinct, electrocyte-specific precursor cells that have been termed ‘electroblasts’ [31, 55]. This is supported by observations in numerous species that the electrocytes never pass through a true muscle fibre stage before maturing—particularly in the electric organ primordia that give rise to Type B electrocytes [1, 31, 45]. Though this contradicts the transdifferentiation observed in *S. macrurus* [41, 61], it has been suggested that transdifferentiation is likely one of multiple mEO regeneration strategies in this species, rather than a requirement of mEO development [55].

### The neurogenic electric organ (nEO)

Instead of a mEO derived from the muscle tissue, members of the Gymnotiform family Apteronotidae possess a nEO derived from the nervous tissue [31, 51, 54]. Limited studies have been performed for the nEO, which is probably due to the fact that it is restricted to a single group within the Gymnotiformes. In addition, for some earlier studies, it is not always clear which member of the Apteronotidae family is being described. As a result, the discussion in this section will be briefer than that of the mEO. The vast majority of the data referred to was obtained from A. *leptorhynchus*.

### The anatomy of the nEO

Like the mEO in other Gymnotiformes, the nEO in members of the Apteronotidae family is located in the tail region, and spans the majority of the length of the body [31]. It consists of a pair of structures that run below and parallel with the spinal cord [44, 54]. Within the abdomen, these structures lie moderately separated, and within the tail, they lie closer together and are separated only by a septum of thin connective tissue, projections of the vertebrae and nerves that run to the skin and ventral muscles [54]. The structures are covered by a sheath of connective tissue, and are distinctly separate to the surrounding muscle [54].

The electrocytes of the nEO are the modified axons of spinal motor neurons, and comprise the entire organ with the exception of associated connective tissue and blood vessels [54]. While the cell bodies of these neurons are located within the spinal cord, the axons descend from the spine into the electric organ below, where they run anteriorly, then sharply turn and run posteriorly until they end blindly at the approximate level at which they entered the organ [44, 54]. Connective tissue strands (bundles of collagen fibres) extend beyond the blind tips of the axons [54]. Where they enter the organ and where they sharply turn, the axons are approximately 20 µm in diameter, and where they run anteriorly and posteriorly, they dilate to a diameter of approximately 100 µm before tapering again. Two types of nodes of Ranvier can be observed in these electrocytes: along the majority of each axon, the nodes have a typical morphology with a gap of about 1 µm in the myelin, and in the distal regions of anteriorly and posteriorly running parts, there are three to five unusually large nodes with gaps of more than 50 µm in the myelin [54].

### The electric discharge of the nEO

Members of the Apteronotidae family, like all Gymnotiformes except *E. electricus*, are weakly electric [31]. While the larval discharge is monophasic (as in the majority of Gymnotiformes), the discharge of the adult nEO is diphasic [54].

As is the case for the mEO, the electric discharge from the nEO represents the summated activity of many electrocytes, which synchronously transform action potentials into external signals. Physiological data indicate that within the electrocytes described above, current propagates along the anteriorly running regions to generate an initial head-positive phase, and the posteriorly running regions to generate a second head-negative phase. Within these regions, it is likely that the typical nodes of Ranvier are active and generate spikes of current, whereas the unusual nodes are inactive and instead act as capacitors (which they are able to do thanks to an extraordinarily large membrane surface area) [54]. However, further details on the mode of action of these neurogenic electrocytes (and how similar it is to that of (a) myogenic electrocytes and (b) typical motor neurons) have yet to be deciphered.

### The development of the nEO

Most developmental data concerning the nEO relate to *A. leptorhynchus*. The development of the electric organ in this species is complicated, with regard to both anatomy and physiology [31]. As mentioned previously, an early myogenic organ first develops, and a neurogenic organ appears only later on in development, proceeding to replace the myogenic one. Larvae begin to exhibit a monophasic discharge at around 9 days post-fertilisation; this initial discharge is the activity of a mEO, and at 11 days myogenic electrocytes can be found in the muscle. At 13 days, additional neurogenic electrocytes begin to develop, and provide the first detectable nEO-derived activity [31]. By the time larvae reach 31 days old, the proportion of the discharge provided by the mEO is quite small, and at 43 days the discharge can be attributed to the activity of the nEO alone [31]. Interestingly, even after the 43-day stage, the form of the discharge continuously changes until fish reach adulthood at about 1 year, at which point they exhibit a typical biphasic discharge. By 162 days, both the number of electrocytes in the nEO and the number of associated motor neurons in the spinal cord are increased [31, 51, 54].

### The evolution of electric organs

After examining what is known about the anatomy, electric discharge and development of the mEO and the nEO within the Gymnotiformes, it is important to consider the ecological factors that are likely to have influenced them, and how they may have evolved.

### Ecological drivers of electric organ evolution

As seen in a variety of animal species, the ecological factors in the living environments of Gymnotiform species would have exerted selection pressures, which led to the evolution of electric organs with traits that enhanced their main functions: electrolocation, electrocommunication, prey incapacitation and predation avoidance [38, 44]. Evidence for the role of these selection pressures tends to be seen more in the discharge of the electric organ than in its anatomy or development.

There is considerable evidence to support the role of predation pressure in shaping electric organ evolution. It has been shown that electroreceptive predators such as *E. electricus* can readily detect low-frequency discharge components from weakly electric fish [46], making predation pressure a plausible explanation for the evolution of high-frequency discharges in prey species [44]. By extension, predation pressure is thought to have been a strong driving force for the evolution of multiphasic discharges, since the addition of phases can increase the frequency of the discharge until it exceeds the sensitivity range of certain predators [46–48].

The presence and characteristics of an electric organ often reflects the lifestyle of the fish possessing it. For example, the nocturnal lifestyle of some species is likely to have led to the preferred use of an electric organ over eyes for navigating the environment and communicating with other individuals. Evidence for this can be seen in all Gymnotiform species: in the eye, both the retina and the lens are smaller than they are in most other fish, which is consistent with its reliance on electrolocation and electrocommunication [1]. Generally, a nocturnal lifestyle also results in the electric organ discharge following a circadian rhythm, exhibiting a greater duration, amplitude and frequency at night when the species’ activity level is the highest [14, 50]. However, this change may incur other costs, as it could make the discharge more detectable to electroreceptive predators such as *E. electricus* and catfish species [46, 48, 56].

Electric organs are hypothesised to play a vital role in reproductive fitness, and evidence for this can be seen in the sexual dimorphism and short-term plasticity of discharge characteristics in the Gymnotiformes [23, 35, 44]. For example, in many species, breeding individuals seem to have discharges with significantly greater durations, amplitudes and baseline frequencies than non-breeding individuals [11, 47, 48, 56, 59]. This likely reflects a temporary prioritisation of attracting mates, competing with other individuals of the same sex and evading predation in order to protect developing offspring [44]. The role of the electric organ in intrasexual competition is particularly well demonstrated by male *B. gauderio*, as the night-time enhancement in discharge duration, amplitude and frequency is increased in the presence of other, competing males, and decreased after a few days of social isolation [14]. Similarly, when exposed to a discharge mimic that simulates that of a competing individual, members of the Apteronotidae family can increase their discharge frequencies by several Hertz, and maintain this for several hours [39].

All animals are subject to the selection pressures exerted by the physical environment that makes up their habitat. For example, waters that are well-oxygenated and fast-flowing (such as those within large, deep river channels) are dominated by the Gymnotiform species with the highest baseline discharge frequencies [10, 11]. It has been proposed that these higher frequencies result in a higher temporal acuity, which would be a distinct advantage in fast-flowing waters, where there would be a large amount of background noise threatening to drown out the signals produced by electric discharges [44].

Since the action potentials generated by the electrocytes are considerably large relative to those generated by typical neurons, it is reasonable to assume that the production of an electric discharge would be energetically costly [44]. The daytime decrease in discharge duration, amplitude and frequency seen in *B. gauderio* is a good strategy for reducing energy expenditure when foraging and reproduction-related activities are not required [22]. A limited energy budget means that high discharge frequencies, which are energetically costly, may come at the expense of other discharge properties, such as amplitude [44]. This particular trade-off can be observed in members the Apteronotidae family [4, 21, 42], and perhaps results in the detection range of a discharge being sacrificed in favour of a higher temporal resolution [44]. When exposed to hypoxia, *E. virescens* and *A. leptorhychus* exhibit a decreased in discharge amplitude (and to a much lesser extent, frequency), demonstrating the use of discharge plasticity for the reallocation of energy from producing the discharge to meeting metabolic demands [43].

### The evolution of the mEO

It has been identified that the first essential step in the process of electric organ evolution must have been the possession of electroreceptors able to sense electric stimuli [25, 33]. Indeed, several species of fish (for example, some catfishes and sharks) have been shown to be highly sensitive to electric fields, but possess no electric organs [13]. It is probable that these electroreceptors are modified receptors of the lateral line system—a system of sensory organs that is conserved among all aquatic vertebrates and whose primary function is to detect movement, vibration and pressure gradients in the water [5, 37]. It is thought that under selection for improved electrolocation, the electrical sensitivity of these electroreceptors increased as certain muscles lost the ability to contract and became specialised for electric discharge production [7]. After the evolution of a weakly electric organ, an increase in the size of the discharge could have increased its value for electrolocation and electrocommunication, until it achieved some limited usefulness as a weapon for prey incapacitation and predation avoidance [7]. Now under selection for these functions to be improved, the organ would then have undergone a further increase in strength and become strongly electric [7]. This sequence of evolutionary events probably occurred convergently in multiple lineages of electric fish.

Studies have investigated the genomic basis of convergent electric organ evolution—specifically that of the mEO in the Gymnotiformes, the Mormyridae and the Malapteruridae [16]. Despite profound morphological differences, the mEOs of these three lineages show common patterns of gene expression, in transcription factors and pathways that contribute to increased cell size, increased excitability and decreased contractility (when compared to normal muscle tissue). This suggests that a common regulatory network may have been repeatedly targeted by selection during the independent evolution of different mEOs—at least in the three lineages investigated [16]. One gene that was found to be highly expressed in all three lineages and which may provide particular insight into mEO evolution is *Scn4aa* [3, 16]. Due to a fish-specific whole-genome duplication that is estimated to have occurred 226–316 million years ago [20], non-electric teleost fish possess two *Scn4a* paralogs that are expressed in the muscle: *Scn4aa* and *Scn4ab* [3]. These code for the voltage-gated sodium channels that are partially responsible for the generation of action potentials [3]. Studies suggest that in electric teleost fish, *Scn4aa* was convergently lost from the muscle but retained in the mEO, before directly contributing to the constructive evolution of the mEO [60]. The latter conclusion is based on evidence that, independently in both the Gymnotiformes and the Mormyridae, (a) *Scn4aa* expression was altered during the origin of the mEO; (b) *Scn4aa* was subjected to extensive positive selection during or immediately after this origin, and (c) this positive selection did not apply to *Scn4ab*, the paralog that remains expressed in muscle [3].

It has been theorised that, in terms of the mEO in the gymnotiformes, Type A electrocytes represent the plesiomorphic condition, which largely stems from the fact that their shape very much resembles that of muscle fibres, which is not the case for Type B electrocytes [31]. This implies that Gymnotiformes possessing Type A electrocytes first arose, after which Gymnotiformes with Type B electrocytes subsequently evolved—a process that would have involved a shift in the location of the electric organ from within the tail muscle to below the tail muscle [31]. The same study also proposed that the electric organs of species retaining Type A electrocytes shifted towards the posterior end of the body during evolution, which can be seen happening on a smaller scale in some species during larval development [31]. In the case of *A. leptorhynchus* (and presumably other members of the Apteronotidae family), a mEO containing Type A electrocytes seems to have been replaced during evolution by a nEO in a more medial part of the body [31].

### The evolution of the nEO

It has been known that the nEOs in the Apteronotidae species are branched out from the spinal cord motor neurons therefore it has been thought that expansion of such neuron has formed the nEO in these species [31, 51, 54]. In species with a mEO, the electric discharge is controlled and coordinated by the ‘pacemaker nucleus’—a group of ‘pacemaker’ and relay neurons in the hindbrain [44]. To activate the mEO, action potentials are produced by pacemaker neurons, then transmitted down the spinal cord by relay neurons to specialised spinal motor neurons, which go on to activate the electrocytes (in the process described earlier) [44]. It has been proposed that the same pacemaker nucleus are connected to the mEO and nEO when both tissues are present, causing similarities between the discharges of the mEO and the nEO in developing *A. leptorhynchus*—specifically, the same frequency [31].

**Fig. 2:**
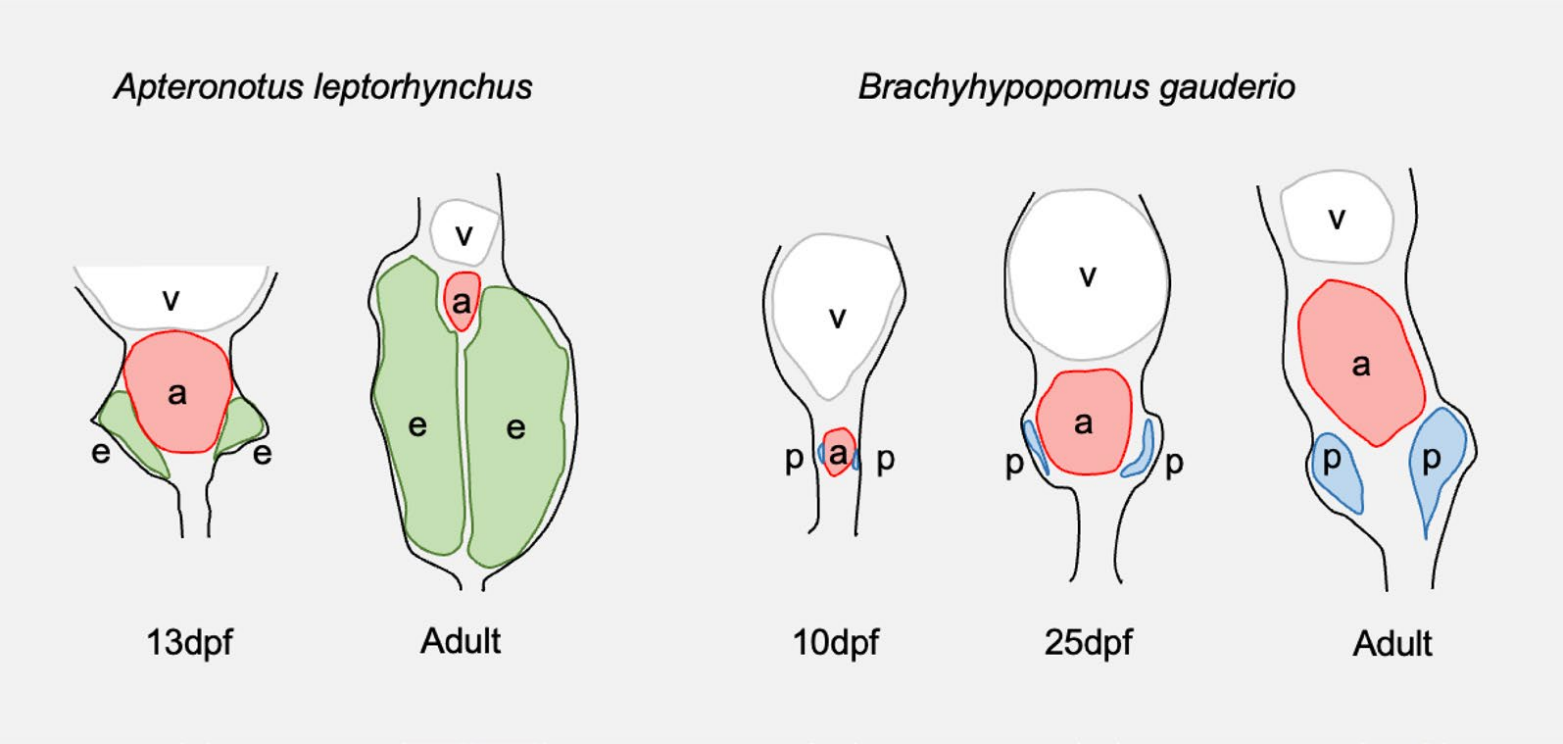
A transverse diagrammatical representation of *Apteronotus leptorhynchus* and *Brachyhypopomus gauderio* at various stages of development. In *A. leptorhynchus*, a neurogenic electric organ (nEO) (e) develops during the larval stage on the ventral side of the vertebrae (v), as two pear-shaped structures located on either side of the aorta (a) [31]. By the adult stage, this nEO has undergone a massive expansion. In *B*. *gauderio*, we have recently discovered that a pair of pear-shaped nerve cords (PSNCs) (p) develop in an equivalent position at the embryonic stage [1]. However, their level of expansion by the adult stage is highly limited compared to that of the nEO in *A. leptorhynchus* [1, 31]. These findings suggest that the PSNCs in *B. gauderio* may represent the evolutionary origin of the nEO in *A. leptorhynchus*

We have recently obtained further supportive evidence from *B. gauderio*: in addition to a mEO, we found a pair of pear-shaped nerve cords (PSNCs) located on either side of the dorsal aorta, with a very similar shape and position to that of the nEO in larval *A. leptorhynchus* [1] (18). These similarities are illustrated by Fig. 2. However, in mature A. leptorhynchus (and other members of the Apteronotidae family), the nEO develops into a very large tissue that occupies the majority of the posterior end of the tail, at which point its morphology no longer resembles that of the PSNCs found in *B. gauderio* [1]. This, again, is illustrated by Fig. 2. These observations suggest that in *B. gauderio*, which may preserve the form of an early Gymnotiform, and in both species, the PSNCs could encompass the motor neurons that regulate the mEO, and during the subsequent evolution of the Apteronotidae lineage, they may have developed and grown large enough to produce a discharge themselves and become a functional nEO, replacing the mEO. During this process, the PSNC-like tissue would have likely undergone a massive expansion as a result of genetic changes, involving both hypertrophy (an increase in cell size) and hyperplasia (an increase in cell number). Were this to be true, the ancestral form of the PSNCs observed in *B. gauderio* could be considered the evolutionary origin of the nEO in the Apteronotidae family.

This hypothesis provides an evolutionary perspective of the Gymnotiformes: the diversification of the mEO and the nEO may have stemmed from an evolutionary branching point in an ancestral species. This species could be an ancestor of *B. gauderio* that possesses mEO and PSNC. In one branch containing the majority of other Gymnotiformes, these PSNCs could have then remained or diminished to leave only a mEO. In another branch containing only the Apteronotidae family, the PSNCs could have instead differentiated to become a fully functional nEO, replacing the mEO which diminished.

To determine the validity of the theory proposed here, a number of steps need to be taken. The PSNCs in *B. gauderio* should be further investigated, in order to determine their connection to both the spine and the mEO, and subsequently the extent to which they regulate the mEO. Other species—both other Gymnotiformes and more distantly related species (electric and non-electric)—should also be looked at to determine whether or not they, too, possess PSNCs.

### Potential Gymnotiform phylogenies

**Fig. 3:**
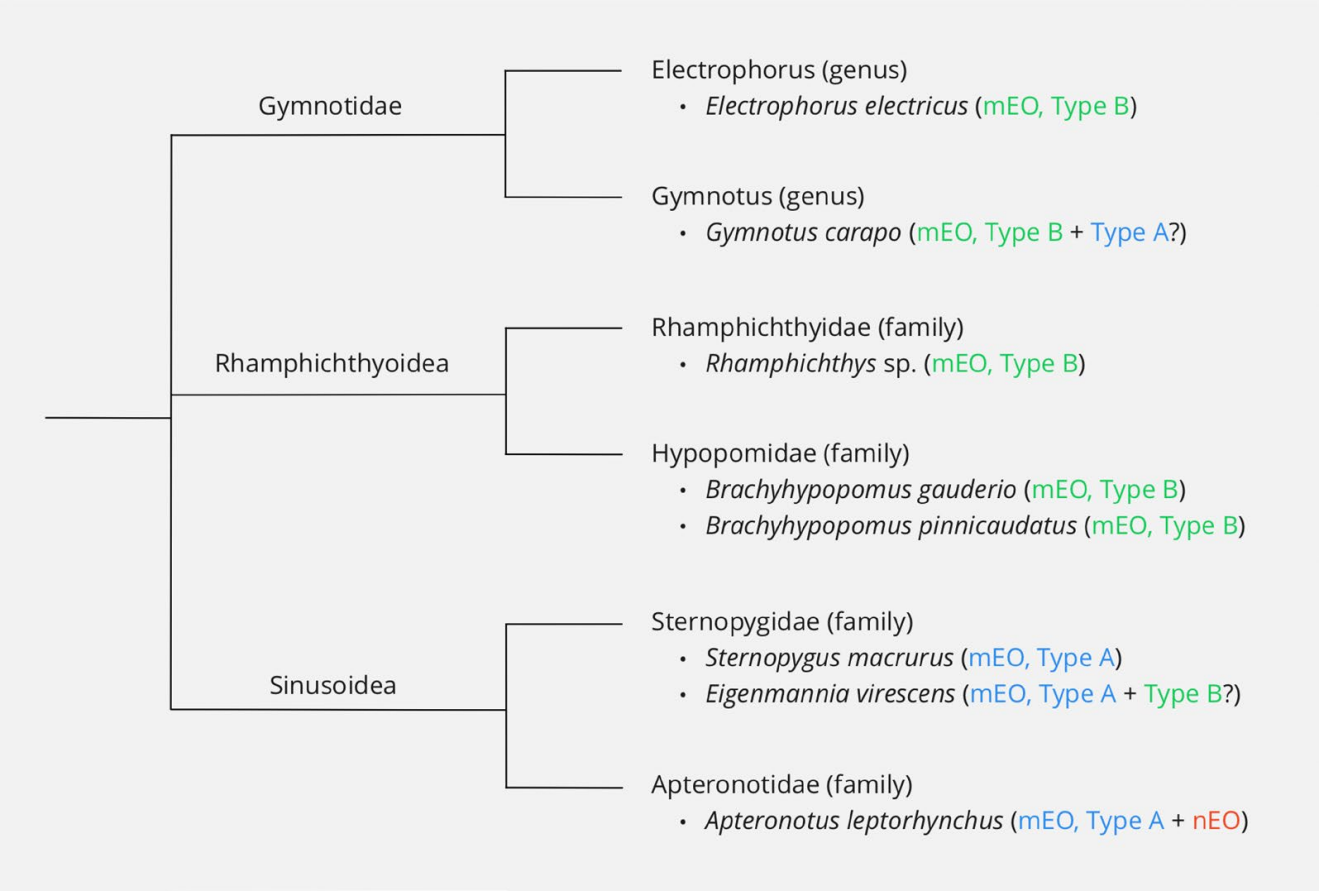
A simplified phylogenetic tree of Gymnotiformes, within which the Gymnotidae, Rhamphichthyoidea and Sinusoidea are considered in parallel. Notable example species are given, along with the corresponding presence of a myogenic electric organ (mEO) with Type A or Type B electrocytes and/or a neurogenic electric organ (nEO) [1, 31]

There are various hypotheses addressing the phylogenetic relationships between different Gymnotiformes [40, 51]. As previously mentioned, three major groups seem to be consistently referred to: the Gymnotidae, the Rhamphichthyoidea and the Sinusoidea [52]. This is illustrated by Fig. 3. Mago-Leccia proposed a phylogeny in which the Sinusoidea lie separated from the Gymnotidae and Rhamphichthyoidea [34]; however, this is not confirmed by molecular and genetic analyses of representative species [52]. Tagliacollo and colleagues later integrated molecular and morphological data to propose an alternative phylogeny, in which the Gymnotidae lie separated from the Rhamphichthyoidea and Sinusoidea [52].

As can be seen in Fig. 3, Mago-Leccia’s proposed phylogeny is supported by the fact that the Gymnotidae and Rhamphichthyoidea tend to possess Type B mEOs, while the Sinusoidea tend to possess Type A mEOs [1, 31]. This grouping together of the Gymnotidae and Rhamphichthyoidea is further supported by similarities (described earlier) in the larval development of Type B electrocytes between *E. electricus*, a member of the Gymnotidae group, and *B. gauderio*, a member of the Rhamphichthyoidea group [1, 45]. Conversely, the hypothesis that the PSNCs in *B. gauderio* represent the evolutionary origin of the nEO in the Apteronotidae family [1] favours a phylogeny more similar to that proposed by Tagliacollo and colleagues, as it would group the Rhamphichthyoidea and Sinusoidea together more closely.

Many theories concerning Gymnotiform evolution are based solely or primarily on morphological traits. Such evidence can be misleading, as morphological traits are often more complex than the categorisation they are given, and similarities can arise through convergent evolution. For example, although Type A and Type B myogenic electrocytes tend to be morphologically and developmentally distinct, more complicated cases are seen in some species [31], as illustrated by Fig. 3. Although *Gymnotus carapo* possesses primarily Type B electrocytes, a small number with partial similarities to Type A electrocytes can be found within the tail muscle [31]. Additionally, although Type A electrocytes develop within *E. virescens* at the larval stage, at a later stage the mEO develops below the tail muscle, in a position similar to that of a typical mEO consisting of Type B electrocytes [31]. To avoid the caveats of a morphology-based phylogeny, further investigation into the developmental pathways of electric organs, in combination with sequencing analysis and modern ‘omics’ technologies (genomics, transcriptomics, proteomics and metabolomics), should be used to deduce the precise evolutionary relationships between different Gymnotiform species. For example, transcriptomics could be used to clarify anatomical conservations between different tissues, particularly the PSNCs in B. gauderio and the nEO in the Apterontidae family.

## Conclusion

In this review, we have discussed what is currently known about the myogenic and neurogenic electric organs of the Gymnotiformes, using numerous example species to demonstrate the diversity present. This diversity is likely due to the combined adaptive responses of Gymnotiform fish to ecological factors such as predation pressure, lifestyle requirements, reproductive competition, habitat characteristics and energetic demands. Numerous theories concerning when and how electric organs evolved have already been proposed, and we make a suggestion that the nEO evolved from a pair of nerve cords that originally functioned to regulate the mEO. Further investigations using conventional methods of studying anatomy and physiology, combined with recent advancements in whole genome research and ‘omics’ technologies, would allow us to further clarify the evolutionary history of electric organs in Gymnotiformes, and other lineages of electric fish.

## Data Availability

Not applicable.
